# Collaborative and Multilingual Approach to Learn Database Topics Using Concept Maps

**DOI:** 10.1155/2014/654397

**Published:** 2014-11-03

**Authors:** Ana Arruarte, Iñaki Calvo, Jon A. Elorriaga, Mikel Larrañaga, Angel Conde

**Affiliations:** University of the Basque Country (UPV/EHU), Manuel de Lardizabal 1, 20018 Donostia, Spain

## Abstract

Authors report on a study using the concept mapping technique in computer engineering education for learning theoretical introductory database topics. In addition, the learning of multilingual technical terminology by means of the collaborative drawing of a concept map is also pursued in this experiment. The main characteristics of a study carried out in the database subject at the University of the Basque Country during the 2011/2012 course are described. This study contributes to the field of concept mapping as these kinds of cognitive tools have proved to be valid to support learning in computer engineering education. It contributes to the field of computer engineering education, providing a technique that can be incorporated with several educational purposes within the discipline. Results reveal the potential that a collaborative concept map editor offers to fulfil the above mentioned objectives.

## 1. Introduction

*A concept map* is a graphical means of illustrating and organising knowledge. It is made up of nodes and links, ordered in such a way so as to reflect the domain information. Nodes symbolize concepts, whereas links show the relationships between concepts. Both nodes and links are labelled and may be categorised. The method of constructing concept maps is known as* concept mapping*. Although concept mapping was originally defined as a means to graphically represent knowledge and information [1], it has since developed into a technique which is useful in both learning and teaching [2].

The fundamentals of concept mapping are in Ausubel's learning theory [3], which is founded on the understanding that meaningful learning takes place when the new concepts are linked to familiar concepts which already exist in the cognitive structure of the learner. Novak and Cañas indicated that one of the arguments for concept mapping being so powerful for promoting meaningful learning is that it acts as a sort of template or scaffold that assists in the organisation and structuring of knowledge [4].

Database systems is an obligatory subject which has to be studied in the second year of the* computer engineering* degree at the* University of the Basque Country*. Along with other topics, the course also consists of the* introduction to database systems*, the entity-relationship model, the extended entity-relationship model, the relational model and relational algebra, and the SQL language. Being a theoretical topic, the* introduction to database systems* is the only area within the subject without practical exercises and has been evaluated traditionally by means of a test. However, this theoretical part is of great importance in order to have sufficient knowledge to understand database systems.

As with many technical subjects, it is essential for database students to learn not only the terminology in their own language but also the English terminology because most of the information sources, technical books, manuals, and web resources are written in English.

The study presented throughout this paper explores the integration of concept maps (CMs) in computer engineering education as a resource for promoting the learning of the subject domain and the acquisition of technical terminology in different languages by means of a creative and collaborative learning experience.

The paper is structured as follows. First, the use of the concept mapping technique in computer engineering studies, in collaborative activities, and in second language learning is reviewed. Next, the design issues of the study and the procedure are described. Finally, the results and discussion are presented.

## 2. Related Work

This section briefly reviews previous work with concept maps in the three pillars that underpin the work presented here: computer engineering discipline, collaborative activities, and second language learning.

### 2.1. Concept Maps in Computer Engineering Discipline

Although they have not been extensively used, some experiences using concept maps in the computer engineering area have been conducted with different educational purposes. Concept mapping has been used as a tool to enhance learning in subjects such as (a) object-oriented programming [5, 6], (b) introduction to computer science [7], (c) parallel computing [8], (d) distributed systems [9], and (e) the I/O subsystem [10, 11].

In addition, this cognitive tool has been used in the identification of key concepts in the engineering design process, in the exploration of expert-student differences [12], and in the organisation of the learning resources related to a subject [13]. Moreover, it has been used as a note taking tool [14].

### 2.2. Concept Maps in Collaborative Activities

Although most of the literature about the use of concept maps in the educational area refers to the use of concept maps by individual students, some attempts to construct concept maps collaboratively have already been carried out. Gao et al. [15] provide a review of studies published after the 1990s in which collaborative concept mapping was used mainly as a learning strategy. Lupion and de Cássia [16] present a survey on the use of concept mapping tools to facilitate collaborative learning in several contexts—e-learning and face-to-face learning—and at different educational levels—preschool, primary, and secondary schools. Kwon and Cifuentes [17] carried out research into the comparative effects of individually and collaboratively built computer-based CMs in middle school learning of science concept. Cañas et al. [18] present LiveMappers.net, a learning environment that supports collaborative concept map-based projects among schools. The results obtained by Layne et al. [19] indicate that CMs are a practical and effective strategy to help distance learners to communicate and collaborate in solving problems in online courses. The cooperation between collaborative learning, concept mapping, and the application of multitouch interactive tabletops is presented in Martínez et al. [20], where a means to track and analyse the flow of knowledge which is created, shared, and gathered when creating a concept map is defined.

### 2.3. Concept Maps in Second Language Learning

There is a scarcity of publicly available studies which look into the validity of concept maps in order to learn terminology in different languages. Some research has been carried out focusing on the following aspects: learning English as a second language [21, 22], working with bilingual concept maps for foreign language vocabulary study [23, 24], and helping students to organise, interact, and share meanings derived from their reading [25]. Concept mapping has also been used as a prewriting strategy [26] and as a form of pretask in the use of tenses in oral accuracy [27].

### 2.4. Previous Experiments

Within the computer engineering curricula, especially in the database area, authors had already taken advantage of the concept mapping approach in two previous works. One of these [28] was focused on the* learning of technical terminology in three languages* (Basque, Spanish, and English). A group of 18 students participated in the experience and the obtained results confirmed a statistically significant difference among the experimental and the control group when learning technical terminology in Basque, Spanish, and English. The other one [29] pursued the engagement of students in the learning subject by means of a* collaborative learning experience*. In this case, 28 students participated in the experience, all of whom were satisfied with the collaborative part of the experience: students pointed out that, throughout the collaboration, they were able to exchange information and knowledge with their peers and also identify new points of view of the same topic. Of course, in the above two cases the learning of the theoretical aspects of subject matter was also a crucial goal.

The new experience, presented in this paper, aims to explore the possibility of working both aspects at once, that is, the learning of technical terminology associated with the area of databases in a nonnative language while developing a concept map collaboratively.

## 3. Study

In the study presented here individual work and collaboration are combined. As the task posed to the students implies different skills—concept mapping, collaboration, and multilingualism—it has been sequenced in order to alleviate the cognitive load of the learners. First, students are asked to draw individually a concept map in their own native language. Then, in the second phase, they collaborate in the construction of the concept map in English. This task sequence allows students to firstly focus on the database domain, learn the basis of concept mapping, and practice with the software used avoiding the difficulties of working in a nonnative language. In the second phase, students face collaboration with peers and work in English.

Next, the design issues, participants, instructional material and learning resources, and procedure of the conducted study are described.

### 3.1. Design

The study occurred throughout the second term of the 2011/2012 academic year in the obligatory* databases* subject of the computer engineering degree at the University of the Basque Country. The database subject is offered during the second course in both Basque and Spanish languages. The subject design and the learning activities that students perform are the same in both languages. As pointed out above, the main goals of the study were the learning of technical terminology associated with the area of databases in the English language while developing a concept map collaboratively.

### 3.2. Participants

36 students divided into two groups participated in the experience; 21 students enrolled in the Basque language group and 15 students enrolled in the Spanish language group. They were enrolled in the subject for the first time and performed the same activities.

Concerning instructors, three lecturers and two Ph.D. students took part in the study. One of the teachers was responsible for the subject, having taught it for 18 years. The other teachers and the Ph.D. students are members of the Ga-Lan research group (http://galan.ehu.es/Galan) of the Computer Languages and Systems Department in the Computer Engineering School at the same university.

### 3.3. Instructional Material and Learning Resources

Each group of students was provided with a 46-slide document which consisted of a summary of the principal concepts and ideas fundamental to the* introduction to databases* topic in their mother tongue (Spanish or Basque) and one bibliographical reference including the first two chapters of the* Fundamentals of Database Systems* book, again in Basque [30] or in Spanish [31]. In addition, a printed copy of the English version of the book was provided to both the Basque and the Spanish groups [32].

**Figure 1:**
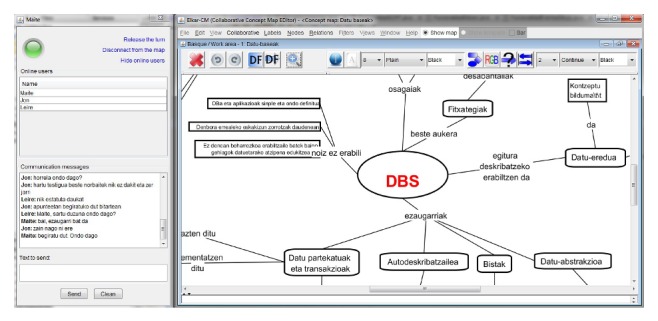
Collaboration process using Elkar-CM.

Regarding the software, the students had Elkar-CM on hand, a collaborative concept map editor implemented in Java (http://galan.ehu.es/Galan/products).  Figure 1 illustrates the interface of Elkar-CM with a concept map on database systems (DBS) written in Basque language. On the right-hand side the edition window, where users can draw the CMs, is displayed. The software offers a wide set of tools to edit the concept map. The operations are included in the menu bar (upper part of Figure 1), in tool bars, and also in contextual menus. Moreover, some frequently used operations (e.g., node resizing, node linkage, etc.) can be performed directly in the central part of the screen. Thus, the users can choose their preferred way of working. On the notepad, located at the bottom of the window, the user writes comments related to a node or relationship. The left-hand side of the figure shows the collaboration management functions: at the top of this window the user can ask or release the token, the connected users are displayed in the middle, and at the bottom (communication messages) the chat service is displayed.

Students were also given handouts which included an introduction to concept mapping, examples of concept maps which were well constructed, instructions to download and install the software, and an online user guide. At first, no human help with the Elkar-CM editor was given to those who participated (only the online user guide).

### 3.4. Procedure

The study consisted of eight phases: (1) content teaching sessions, (2) study presentation and call for participation, (3) Elkar-CM presentation, (4) individual concept map development, (5) student team formation and collaborative concept map development, (6) survey, (7) exam, and finally (8) analysis and evaluation of the concept maps, logs, tests, and exams.

*Content Teaching Sessions*. Students also participated in an introductory 90 min lecture on the chosen topic. This time, students were only provided with the 46-slide summary which contained the principal ideas concerning the topic.

*Study Presentation and Call for Participation*. In the 30 min lecture, the subject lecturer briefly explained the main characteristics of the study to the students, all of whom agreed to participate in the experience. It must be mentioned that all of the students who enrolled in the subject had chosen to follow a formative evaluation approach instead of a summative evaluation approach which was also available in the subject. The instructors gave the students handouts which contained important details such as specific activities to complete and timetable. The students were also given an introduction (15 min) to the strategy of concept mapping and they were given examples of well-constructed CMs.

*Elkar-CM Presentation*. During the first 15 min of a 1 and 1/2 hours' laboratory session, the students were briefly given an introduction to the principal characteristics of Elkar-CM for individual work. Next, they were given the opportunity to practice with the tool and started creating a CM related to the topic of database systems. Also, the students were given instructions on how to download and install the software on their own computers.

**Figure 2:**
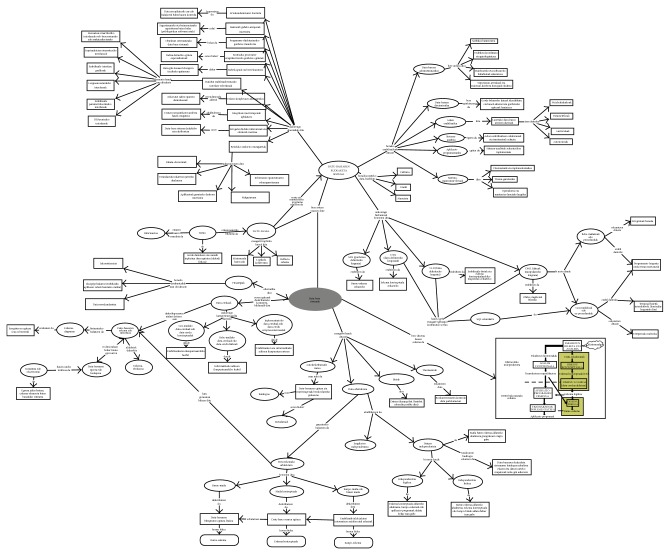
Concept map with the largest number of domain topics.

**Figure 3:**
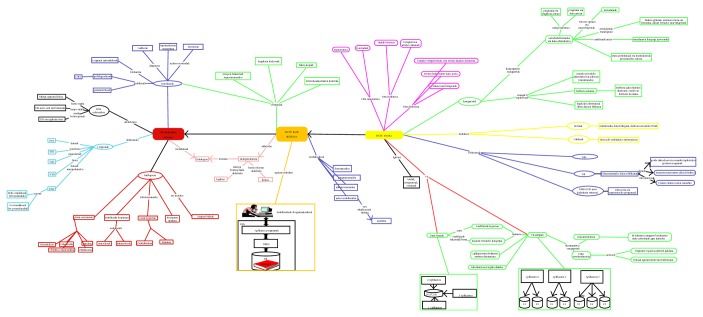
Concept map that prioritizes the visual appearance.

*Individual Concept Map Development*. Before submitting the map to the instructors, the students had three weeks to work on it, throughout which time they worked autonomously completing their CM which they had started in the lab session. The subject teacher and the software instructors were available for the students whenever they needed assistance. Figures 2 and 3 show two individually created CMs. Although the text of the labels in both figures is unreadable, they show two clearly different ways of structuring and organising the subject topics. While the map shown in Figure 2 aims at representing the largest number of domain topics, the concept map in Figure 3 prioritizes the visual appearance of the map. In this last case, the student organised the domain topics using some of the visual resources, such as colours or images, provided by the tool.

*Student Team Training and Collaborative Concept Map Development*. The students were given a quick introduction to the main collaborative characteristics of Elkar-CM in a 90 min lab session: token-passing mechanism, chat, notes area, and so forth. Previous experiences with Elkar-CM indicated that two or three people are the optimum sizes for working such a type of collaborative concept mapping [29]. Therefore, they were organised in teams—twelve teams of two people and four teams of three people. In each working team, students belong to the same language group so they can communicate in their own language through the chat mechanism provided by Elkar-CM. Each student had the images of the concept maps the team members created in the individual phase. Working teams were previously established by instructors following the alphabetical order criteria and, after the members of each team had been separated into two different laboratories, they collaboratively created a CM from the beginning. Again, the database systems was the central topic but, this time, students were asked to create the CM in the English language. After the session the information saved in the server—the final English version concept maps, the information about the collaborative concept mapping process along with the interaction logs—was collected by the instructors in order to be evaluated.

*Survey*. Each student completed a survey to evaluate the experience. The questionnaire was composed of 22 closed items and 6 open items. The closed items were Likert-type questions, whose answers were adjusted to a four-point scale (4: “good,” 3: “satisfactory,” 2: “unsatisfactory,” and 1: “bad”). The questionnaire provided the opportunity for the participants to give their opinion about the following points: the interest the student had in the study, prior experience with concept maps in general and with concept mapping software, differences between working individually or collaboratively when learning the subject learning, quality of the created concept maps, how useful the concept mapping technique was for learning purposes in general and, specifically, for learning database concepts and terminology in English, value of the collaboration and interaction which took place during the activity, appropriateness of the features of Elkar-CM, and simplicity of use of Elkar-CM. Students were also asked to report problems encountered during the study.

*Exam.* In the exam, 5 points (out of 10) were related to the introduction to database systems topic.

*Analysis and Evaluation of the Concept Maps, Logs, Tests, and Exams*. With the objective of analysing the final results, the subject lecturer worked together with the software developers (see next section).

## 4. Results and Discussion

Five sources of data have been analysed to evaluate the results of the study: individually created concept maps, collaboratively created concept maps, interaction history when creating the concept maps collaboratively, students' responses to the survey, and students' marks in their exams.

To evaluate the* individually generated concept maps*, the subject matter teacher analysed the topic completeness and thoroughness of each final CM. Three levels of performance were established: good (2), satisfactory (1), and unsatisfactory (0). The majority of the students (69%) got the highest mark and only 13.7% failed. The average number of nodes created by each student was 83.1% and the average number of relations was 20.5%.

The average number of nodes and relationships established in the* collaborative created concept maps* was smaller than in the individual ones: an average of 61 nodes per concept map and 10 relationships. It must be taken into account that the students had three weeks for creating the individual concept maps and one hour for creating the collaborative concept maps. Nevertheless, when evaluating the concept map completeness and thoroughness of the collectively created concept map, none of them was evaluated as unsatisfactory. It seems that although the teams were formed using an arbitrary procedure, alphabetical order between the members of each language group, the results were beneficial. In all those teams in which the individual map generated by one of its members was evaluated as unsatisfactory, the collaboratively created concept map was positively evaluated.

Another important aspect that has been analysed in the collaboratively created concept maps is the* quality of the English* language used. Although individual concept maps were created in the student's mother tongue, the collaboratively created concept maps must be labelled using the English language. Remember that one of the aims of this study was to promote the learning of database related terminology in English. To reach this objective in the collaborative laboratory session, students were provided with* Fundamentals of Database Systems* in English [32]. The high linguistic accuracy of the English labels in all the concept maps is worth mentioning. Students deeply analysed the provided learning material looking for the appropriate translation of the labels they identified in their individual concept maps.

Taking into account that the English concept maps were created collaboratively, instructors considered it important to analyse the* students' interaction history*, that is, the process of how activities were carried out.* Chat logs* are the data source used in this analysis.

All the analysed chats had three clear phases. In the first one, participants introduced themselves and planned the task. In fact, one of the first decisions they had to take was to determine whether or not they would use one of the conceptual maps created in the individual phase and which concept map would be the starting point. 87.5% of the teams decided to use a previous concept map and translate it. Only two teams, one in each language, preferred to create the collaborative concept map from scratch. However, these two teams clearly distributed the work to be done: one member would focus on Chapter 1 and the other on Chapter 2 [32].

The longer phase of the chat, the second phase, focused on the process of creating the concept map. The main activity was related to label translation issues. In addition to using the two chapters of the book in English, the chat analysis revealed that at least five of the teams (31.3%) also used online dictionaries and language translators.

Finally, the last phase of the chat was devoted to congratulating each other on the work done and to the finishing of the task.

The collaborative learning conversation skills taxonomy developed by Soller [33] includes three learning conversation skill types: conversation, active learning, and creative conflict. Once the interactions obtained from the logs of the chat were classified into the main skills identified by Soller, it could be observed that 28.6% (first and third phases) were related to* conversation* and 71.4% (second phase) to* active learning* and* creative conflict.*

Table 1 summarizes the chat intervention percentage in each phase during the collaborative concept map creation process. On average teams performed almost 100 chat interventions.

Concerning the* surveys or questionnaires conducted by students*, their analysis has led to some interesting conclusions. For example, it is clear that almost all of the students had very little previous experience with the concept mapping technique and none of them had ever used concept mapping software.

As regards the interest in participating in the experiment, most students were highly motivated. Moreover, the greater the time spent on completing the individual concept map, the higher the valuation that students had about the quality of their own concept map. This appreciation is backed by the teacher's assessment of the individually drawn concept maps and the results of the exam. The concept maps made by the members of the students who spent more time working on the drawing got higher marks in both the concept map and the exam. By contrast, in the case of the collaborative activity, all the students spent the same time and it can be observed that the personal assessment does not differ between them. In a similar way, all the students believed that the interaction that was maintained while doing the collaborative map was appropriate.

The answers to the questions related to the improvement of the students' knowledge on the subject matter indicate that almost all the students positively valued the knowledge they acquired during this activity. Performing the activity helped them in working on the topics of the course.

All the participants valued the collaborative activity more positively than the individual one. In addition, they suggested that it would have been beneficial to work on this collaborative activity from home with extra time.

Again, all the students agreed that they found the concept mapping technique adequate for both learning the subject domain and learning the terminology in other languages. In addition, students said Elkar-CM is easy to use concept mapping software.

Finally, with reference to the* exam*, it was divided into two parts; the first part comprised questions on the topics that are worked on in the experiment, whereas the second part entailed questions about other issues. Results confirm that a bigger number of students passed the questions related to the topics that are worked on in the concept map experience.  Table 2 shows the number and percentage of students who passed each part of the exam. In addition, students got one point, on a 0 to 10 scale, more in the questions related to the topic that is worked on in the study. A paired Student's *t*-test confirms that results are statistically significant (*P* value = 0.0359563 < 0.05).  Table 3 shows the average marks in both groups of students.

In regard to the interest in participating in the experiment, all students enrolled in the Basque group were highly motivated, while the motivation of the students enrolled in the Spanish group was lower. This fact had a direct impact on the number of hours spent on carrying out the individual activity. Although the average number of hours was 2.4, the distribution between the Basque and the Spanish group was 3 hours and 1.8 hours, respectively. Moreover, the greater the time spent on completing the individual concept map, the higher the valuation that students had about the quality of their own concept map. This appreciation is backed by the teacher's assessment of the individually drawn concept maps and the results of the exam. The concept maps made by the members of the students who spent more time working on the drawing (Basque group) got higher marks in the concept map. In addition the students who were more motivated got considerably higher marks in the first part of the exam than in the second one (see Tables 2 and 3).

Concerning gender, the sample was not balanced; this is also the reality when considering all the students registered in computer engineering degrees. There were a minority of women (25%). In the Basque group, 23.81% of the participants were women; meanwhile in the Spanish group the percentage of women was a little bit higher (26.66%). Consequently, it was not possible to get significant results regarding gender differences.

## 5. Conclusions

In this paper, a study that combines individual work and collaboration using a concept mapping software in computer engineering education had been presented. The concept mapping technique had been incorporated with different educational purposes within the computer engineering discipline, especially in the* database systems* subject.

The task the students were asked to carry out implied several skills: theoretical database knowledge, concept mapping, collaboration, and technical terminology learning. First, students were asked to draw individually a concept map in their own native language on database topics. Next, they collaborated in the construction of a concept map in English. This task sequence allowed students to firstly focus on the database domain, learn the basis of concept mapping, and practice with the software used avoiding the difficulties of working in a nonnative language. In the second phase, students faced collaboration with peers and work in English.

The results obtained in the study—concept maps quality and exam marks—were positive. With regard to the learning of the English technical terminology, the accuracy of the use on the English language in the collaboratively created concept maps was analysed by instructors.

As regards exams, results confirm that a bigger number of students passed the questions related to the topics that are worked on in the concept map experience. In addition, students who participated in the experience got a bigger mark in the questions related to the topic that is worked on in the study than in those not related to it.

It is also remarkable that all the students invited participated in the experiment. Even those students who were not highly motivated to participate in the study stated that their participation enabled them to learn and enter deeply into a technique for both learning the subject terminology in the English language and learning the contents of the subject domain.

Another important aspect worth mentioning is the preference of the collaborative task over the individual one. Although virtually all students recognized that the concept maps created individually were more complete than those drawn collaboratively, they preferred the collaborative activity where they were able to share and exchange ideas with classmates.

The analysis of the chat interaction enabled instructors to study the way students perform the tasks and the problems they encountered. It is also useful to improve the setting of the task in future experiments, with better planning and an appropriate learning context.

Pursuing the objective that in the near future students belonging to linguistically different groups could participate in the collaborative activity, Elkar-CM designers aim to integrate translators and dictionaries in the tool language that facilitate the communication process between linguistically or culturally distinct groups of people.

## Figures and Tables

**Table 1:** Chat intervention percentage in each phase.

	Phase I	Phase II	Phase III
Interventions	20,19%	71,37%	8.42%

**Table 2:** Number and percentage of students that passed each part of the exam.

	Pass			
	Part I		Part II	
	Number	Percentage	Number	Percentage
Basque	19	90.47%	11	52.38%
Spanish	12	92.30%	9	69.23%
Global	31	91.17%	20	55.55%

**Table 3:** Average mark (0 to 10 scale) in each part of the exam.

	Average mark part I	Average mark part II
Basque	6.49	5.45
Spanish	6.39	5.35
Global	6.44	5.41
